# Socioeconomic inequalities in disease prevalence by age and sex for 17 common long-term conditions in England: retrospective, observational study of electronic primary care records from Clinical Practice Research Datalink (CPRD) Aurum

**DOI:** 10.1136/jech-2024-223553

**Published:** 2025-07-29

**Authors:** Nils Gutacker, David Glynn, Anne Mason, Simon Mark Walker, Luigi Siciliani, Tim Doran

**Affiliations:** 1 Centre for Health Economics, University of York, York, UK; 2 Department of Economics and Related Studies, University of York, York, UK; 3 Health Sciences, University of York, York, UK

**Keywords:** Health inequalities, EPIDEMIOLOGIC MEASUREMENTS, GENERAL PRACTICE

## Abstract

**Background:**

Evidence on socioeconomic inequalities in the prevalence of common long-term conditions and their variation across the life course is necessary for equitable service design and resource allocation. We used routinely collected electronic primary care records and a unified data extraction and analysis framework to estimate socioeconomic variations in the prevalence of 17 common long-term conditions by age and sex.

**Methods:**

Electronic records for 2.2 m patients registered with 300 randomly selected primary care practices contributing to the Clinical Practice Research Datalink Aurum database were used to estimate observed, age-sex standardised and age-specific rates of disease prevalence on 31 March 2020 by Index of Multiple Deprivation quintile groups. Inequality in disease burden was expressed as the prevalence rate ratio (RR) between the most and least deprived fifths of the population.

**Results:**

Age-sex standardised prevalence rates were higher in the most deprived compared with the least deprived fifth of the population for 16 of 17 conditions. The largest relative differences in disease prevalence were observed for chronic obstructive pulmonary disease (RR: 3.29; 95% CI: 3.19 to 3.38), severe mental illness (RR: 2.72; 95% CI: 2.60 to 2.85) and peripheral arterial disease (RR: 2.58; 95% CI: 2.46 to 2.72). For most conditions, the equity gap was largest in middle age and reduced with age thereafter.

**Conclusions:**

Substantial socioeconomic inequalities in disease prevalence are evident in the English population. A catalogue of disease prevalence by socioeconomic quintile group, age and sex is provided to facilitate further analysis and modelling.

**WHAT IS ALREADY KNOWN ON THIS TOPIC**

- Socioeconomic inequalities in the prevalence of long-term conditions are well recognised, but previous studies have often lacked age-specific and sex-specific detail or standardised methodologies. This has limited understanding of how inequalities vary across the life course and between conditions, hindering equitable service planning.

**WHAT THIS STUDY ADDS**

- We calculated standardised and age-sex-specific estimates of prevalence for 17 common long-term conditions in England by socioeconomic deprivation.
- For most conditions, prevalence increases with deprivation, but there is marked variation between conditions and by sex.
- Relative socioeconomic inequalities in prevalence tend to peak in middle age before declining in older age.

**HOW THIS STUDY MIGHT AFFECT RESEARCH, PRACTICE OR POLICY**

- Our findings offer a reference dataset for modelling health needs and service use across demographic and socioeconomic groups, facilitating more precise and equitable targeting of public health resources.

## Introduction

 Socioeconomic inequalities in health are a persistent global issue,^1^,^3^ with lower socioeconomic groups experiencing higher prevalence and worse outcomes for a range of long-term conditions.^4^,^6^ Addressing this issue requires robust estimates of disease prevalence disaggregated by equity relevant variables to identify inequalities, target interventions and allocate resources fairly.^7 8^ Obtaining such estimates relies on accurate and consistent identification of cases linked to robust measures of socioeconomic status.

Electronic primary care records (EPCRs) contain detailed patient information, including comprehensive medical histories, clinical measurements, prescriptions and demographic data.^9^ Data are collected prospectively for the purpose of case management using existing clinical data infrastructures, hence EPCRs are an efficient source of longitudinal data for estimating disease prevalence over time, with relatively low risk of selection and information bias, particularly for rare conditions.^10^ In England, most people are registered with a general practice which provides primary care services, acts as a gatekeeper for non-urgent secondary care and has a coordinating role in active disease management. EPCRs can, therefore, provide more representative estimates of disease prevalence than national health surveys and more nuanced and complete prevalence estimates than mortality data.

Previous estimates based on English EPCRs have established that the prevalence of common cardio-renal-metabolic and mental health conditions generally increases with socioeconomic deprivation and age.^11^,^13^ However, overall prevalence estimates underestimate inequalities for some age groups and overestimate them for others, as most long-term conditions are not only more prevalent in more deprived populations, they develop at an earlier age,^2^ hence health inequalities vary substantially by age group.^14 15^ The nature of these interactions is likely to vary by disease, and so there is a need to map variations in prevalence for different combinations of age, social group and condition.

Inequalities for a given condition also need to be understood in terms of both observed (raw) and age-sex-adjusted prevalence rates. The former measure is useful for healthcare providers and planners in understanding the burden of disease for different socioeconomic groups within a population and recognises that different socioeconomic groups have different age-sex structures. The latter measure relates to an individual’s risk of developing a condition based on their socioeconomic status and gives an indication of how ‘fair’ the distribution of disease is.

In this study, we linked EPCRs to area-level deprivation at the individual patient level to estimate socioeconomic variation in the prevalence of 17 common long-term conditions. In addition, we: (a) report age-specific and sex-specific rates of disease prevalence by deprivation quintile group, which permits us to explore variation in inequalities over the life course and (b) compare observed prevalence rates with directly age-sex-standardised rates.

## Methods

### Choice of condition

We examined the recorded prevalence of 17 long-term conditions included in the Quality and Outcomes Framework (QOF), a national pay-for-performance scheme. The QOF requires primary care practices to maintain disease registers for common long-term conditions and provides financial incentives for meeting a range of quality-of-care targets.^16^ We focused on single QOF conditions, or clusters of closely related conditions, and restricted our analysis to conditions for which QOF requires registration of all adults (table 1). The scheme provides strong incentives for practices to case find, as quality payments scale with disease prevalence, and disincentives to over-report prevalence, as payments are contingent on the delivery of appropriate care to patients with the relevant conditions. The QOF scheme also requires practices to document diagnoses and clinical management activities using EPCR systems with strict interoperability requirements^17^ using a standard set of clinical codes, reducing the potential for misclassification and facilitating consistency in recording.^18^

**Table 1:** List of long-term conditions and QOF requirements

Code	Disease or disease group	Age range of QOF register	Additional requirements imposed by the QOF
AST	Asthma	All	A patient has received asthma-related drug treatment in the last 12 months.
AF	Atrial fibrillation	All	
CAN	Cancer	All	A patient has had a first or new diagnosis of cancer on or after 1 April 2003.
CHD	Chronic heart disease	All	
CKD	Chronic kidney disease	18+	A patient has chronic kidney disease with classification of categories G3a to G5.
COPD	Chronic obstructive pulmonary disease	All	
DEM	Dementia	All	
DEPR	Depression	18+	A patient has had a first or new episode of depression on or after 1 April 2006 that has not resolved since.
DM	Diabetes mellitus	17+	
EP	Epilepsy	18+	A patient has received epilepsy-related drug treatment in the last 6 months
HF	Heart failure	All	
HYP	Hypertension	All	
MH	Severe mental illness (psychosis, schizophrenia or bipolar affective disorder)	All	
OB	Obesity	18+	A patient has had a measured BMI of 30 or over at any point in the last 12 months
PAD	Peripheral arterial disease	All	
RA	Rheumatoid arthritis	16+	
STIA	Stroke and transient ischaemic attack	All	

BMI, body mass index; QOF, Quality and Outcomes Framework.

### Data source and sampling procedure

Anonymised EPCRs for patients registered in English primary care practices were extracted from the Clinical Practice Research Datalink (CPRD) Aurum. The CPRD Aurum dataset collates patient-level information on diagnoses, symptoms, referrals, tests and prescriptions from participating practices that use EMIS Web software. As of April 2020, CPRD Aurum included approximately 32 million patients currently or previously registered with over 1200 participating practices. CPRD Aurum is broadly representative of the English population in terms of age, sex and deprivation,^19^ but participating practices are more likely to be located in urban areas.^20^

Our sample was determined using a two-stage process. First, a random sample of 300 practices was selected from the pool of 1166 practices contributing to CPRD Aurum on 31 March 2020 (the index date). Second, clinical records were extracted for all patients that met the inclusion criteria: (1) aged 18 or over on the index date; (2) registered with a sampled general practitioner (GP) practice and (3) with a recorded diagnosis of one or more of the 17 conditions of interest at any time (table 1).

For each patient, CPRD Aurum provides information on age, sex and the deprivation profile of the neighbourhood in which they resided on the index date. The latter is based on the Index of Multiple Deprivation (IMD),^21^ which ranks all lower-layer output areas (LSOAs) in England according to a weighted composite indicator covering seven domains: income; employment; education, skills and training; health and disability; crime; housing; and living environment. LSOAs are assigned to quintile groups based on their rank, and these quintile groups were mapped to patients’ postcode information by the data provider.

### Statistical analysis

We calculated point prevalence rates by IMD quintile group for each study condition on 31 March 2020. Prevalent cases were identified using the disease definitions and clinical code lists of the English QOF business rules for the financial year 2019/2020 (v44.0). Patients were deemed to have the condition if a relevant diagnosis code had been recorded at any point in time and no ‘disease resolved’ code was issued subsequently to the last diagnosis recording. For some conditions, additional restrictions were applied in the business rules (table 1). The denominator includes all patients aged 18 or over and registered with a sampled GP practice on the index date. CPRD provided total counts of patients by 5-year age bands, sex and IMD quintile group for all practices. The denominator data are not disaggregated by practice to avoid potential reidentification of patients or practices due to small cell counts.

We calculated sex-specific prevalence rates by IMD quintile for each 5-year age band. Rates for each 5-year age-sex-IMD quintile cell were then multiplied by the proportion of the English standard population (2020 mid-year estimates) in each age-sex group calculated either (1) within each IMD quintile (approach A) or (2) across the entire population (approach B), and then summed to produce population prevalence rates by IMD quintile. Approach A results in prevalence rates for the five population subgroups that maintain observed differences in age-sex composition across socioeconomic groups in the population of England. Our calculations thus serve to adjust for any imbalance in the age-sex distribution within our sample and the general population in the corresponding IMD quintile groups. Approach B results in directly standardised prevalence rates for five hypothetical populations of identical age-sex composition that differ only in their level of deprivation.

Prevalence rates were expressed as percentages (ie, cases per 100 patients at risk), with approximate 95% CIs derived using the Poisson method.^22^ Multimorbid patients contributed to more than one prevalence analysis.

Inequality was measured as prevalence rate ratios (RRs), calculated by dividing prevalence rates in the most deprived quintile group (Q1) by rates in the least deprived quintile group (Q5). Values above 1 indicate higher rates in the more deprived group of patients. We also report absolute gaps in prevalence rates between Q1 and Q5. Ratio measures were selected over alternative measures of inequality, such as slope or relative indices of inequality (SII, RII), due to their ease of interpretation when comparing across conditions.

## Results

2 194 606 patients aged 18 or over were registered with sample practices on 31 March 2020. Of these, 894 794 (40.8%) had at least one study condition, and 417 027 (19.0%) exhibited multimorbidity (more than one study condition).

### Observed and age-sex-standardised prevalence rates

Table 2 provides estimated disease prevalence rates in the most deprived (Q1) and least deprived (Q5) quintile groups as well as inequality gap measures (approach A). Estimates of SII and RII by condition, overall prevalence rates and rates for IMD quintile groups 2–4 are reported in the online supplemental material. Age-sex-standardised prevalence rates for study conditions ranged from 0.76% (95% CI: 0.75% to 0.79%) for epilepsy to 18.03% (95% CI: 17.97% to 18.09%) for hypertension. There was no clear socioeconomic pattern in disease prevalence: 10 of 17 conditions had significantly higher prevalence in the most deprived patient populations, including three conditions with prevalence rates of more than 5% in the adult English population: diabetes (RR: 1.47; 95% CI: 1.45 to 1.50), depression (RR: 1.53; 95% CI: 1.51 to 1.55) and obesity (1.63, 95% CI: 1.60 to 1.65). Five conditions had significantly lower prevalence in the most deprived patient populations, and for two conditions, there was no statistically significant difference in rates between the most and least deprived quintile groups.

**Table 2:** Inequality in observed prevalence rates between most and least deprived population quintile (approach A)

Disease group	IMD1		IMD5		Relative inequality		Absolute inequality	
	Rate	95% CI	Rate	95% CI	RR	95% CI	Difference	95% CI
Atrial fibrillation	2.16%	(2.11% to 2.20%)	3.23%	(3.17% to 3.29%)	0.67	(0.65 to 0.69)	−1.07%	(−1.15% to −1.00%)
Asthma	6.53%	(6.45% to 6.61%)	6.17%	(6.09% to 6.25%)	1.06	(1.04 to 1.08)	0.36%	(0.25% to 0.47%)
Cancer	3.32%	(3.27% to 3.38%)	5.38%	(5.31% to 5.45%)	0.62	(0.60 to 0.63)	−2.06%	(-2.15% to −1.96%)
Chronic heart disease	4.19%	(4.12% to 4.26%)	3.85%	(3.79% to 3.92%)	1.09	(1.06 to 1.11)	0.33%	(0.25% to 0.42%)
Chronic kidney disease	4.11%	(4.04% to 4.17%)	4.84%	(4.78% to 4.91%)	0.85	(0.83 to 0.87)	−0.74%	(−0.83% to −0.64%)
Chronic obstructive pulmonary disease	3.80%	(3.73% to 3.86%)	1.69%	(1.65% to 1.74%)	2.24	(2.18 to 2.31)	2.10%	(2.03% to 2.18%)
Dementia	0.98%	(0.95% to 1.02%)	1.07%	(1.04% to 1.10%)	0.92	(0.88 to 0.96)	−0.09%	(−0.13% to −0.04%)
Depression	16.98%	(16.85% to 17.10%)	11.10%	(11.00% to 11.20%)	1.53	(1.51 to 1.55)	5.88%	(5.71% to 6.04%)
Diabetes mellitus	8.67%	(8.58% to 8.77%)	5.89%	(5.81% to 5.97%)	1.47	(1.45 to 1.50)	2.78%	(2.66% to 2.90%)
Epilepsy	1.01%	(0.98% to 1.04%)	0.65%	(0.62% to 0.67%)	1.56	(1.48 to 1.64)	0.36%	(0.32% to 0.40%)
Heart failure	1.34%	(1.31% to 1.38%)	1.15%	(1.12% to 1.19%)	1.17	(1.12 to 1.21)	0.19%	(0.14% to 0.24%)
Hypertension	17.36%	(17.23% to 17.49%)	18.48%	(18.35% to 18.62%)	0.94	(0.93 to 0.95)	−1.12%	(−13.11% to −0.93%)
Severe mental illness (psychosis, schizophrenia or bipolar affective disorder)	1.62%	(1.58% to 1.66%)	0.63%	(0.61% to 0.66%)	2.56	(2.45 to 2.68)	0.99%	(0.94% to 1.04%)
Obesity	12.98%	(12.86% to 13.09%)	7.98%	(7.90% to 8.07%)	1.63	(1.60 to 1.65)	4.99%	(4.85% to 5.13%)
Peripheral arterial disease	1.07%	(1.04% to 1.10%)	0.63%	(0.60% to 0.65%)	1.70	(1.62 to 1.79)	0.44%	(0.40% to 0.48%)
Rheumatoid arthritis	0.84%	(0.81% to 0.87%)	0.82%	(0.80% to 0.85%)	1.02	(0.97 to 1.07)	0.02%	(−0.02% to 0.06%)
Stroke and transient ischaemic attack	2.31%	(2.26% to 2.36%)	2.37%	(2.32% to 2.41%)	0.98	(0.95 to 1.01)	−0.05%	(−0.12% to 0.01%)

IMD, Index of Multiple Deprivation; RR, rate ratio.

**Figure 1:**
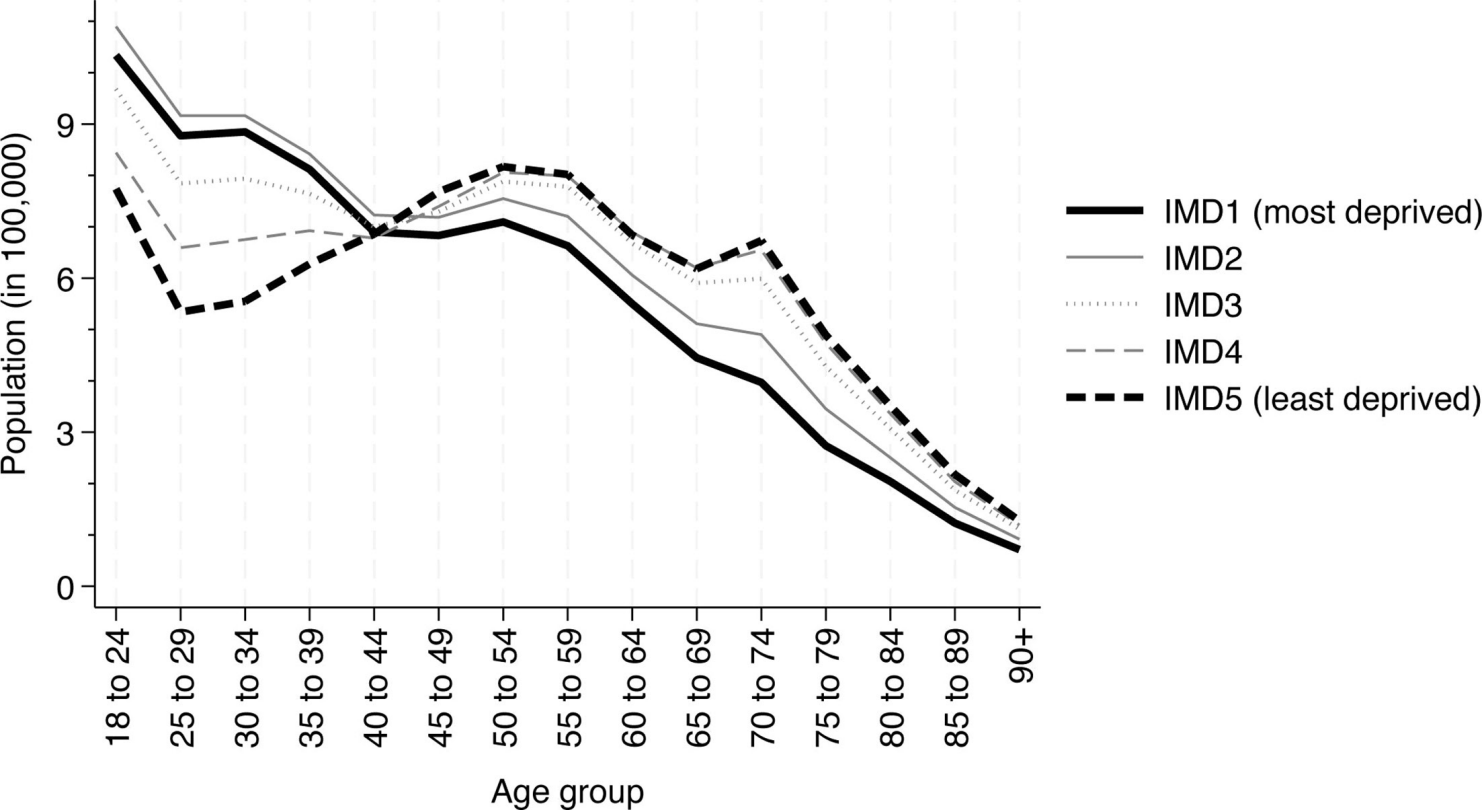
English population in 2019 by age group and deprivation quintile. IMD, Index of Multiple Deprivation. Source: Office for National Statistics.^23^

There are marked differences in the age profile of the different deprivation quintile groups in England with more deprived groups being younger on average^23^ (see also figure 1). These differences in age structure may combine with differences in disease prevalence by age group to disguise inequalities in overall disease prevalence across socioeconomic groups. Table 3 shows prevalence rates by deprivation group after direct standardisation to a common age-sex distribution (approach B). Following standardisation, 16 of 17 conditions had statistically significantly higher age-sex standardised prevalence rates in the most deprived fifth of the population compared with the least deprived fifth. The largest relative differences in disease prevalence were observed for chronic obstructive pulmonary disease (COPD) (RR: 3.29; 95% CI: 3.19 to 3.38), severe mental illness (RR: 2.72; 95% CI: 2.60 to 2.85) and peripheral arterial disease (RR: 2.58; 95% CI: 2.46 to 2.72). Only cancer (RR: 0.87; 95% CI: 0.85 to 0.89) had significantly lower prevalence rates in the most deprived fifth of the population. The largest differences in absolute disease prevalence were observed for obesity (6.50%; 95% CI: 6.35% to 6.64%), depression (5.80%; 95% CI: 5.64% to 5.97%) and hypertension (5.00%; 95% CI: 4.80% to 5.20%).

**Table 3:** Inequality in age-sex standardised prevalence rates between most and least deprived population quintile (approach B)

Disease group	IMD1		IMD5		Relative inequality		Absolute inequality	
	Rate	95% CI	Rate	95% CI	RR	95% CI	Difference	95% CI
Atrial fibrillation	2.79%	(2.73% to 2.85%)	2.68%	(2.63% to 2.73%)	1.04	(1.01 to 1.07)	0.11%	(0.03% to 0.19%)
Asthma	6.85%	(6.76% to 6.93%)	5.99%	(5.91% to 6.06%)	1.14	(1.12 to 1.16)	0.86%	(0.74% to 0.97%)
Cancer	4.04%	(3.97% to 4.11%)	4.63%	(4.57% to 4.70%)	0.87	(0.85 to 0.89)	−0.60%	(−0.69% to −0.50%)
Chronic heart disease	5.25%	(5.17% to 5.33%)	3.22%	(3.17% to 3.27%)	1.63	(1.59 to 1.67)	2.03%	(1.93% to 2.13%)
Chronic kidney disease	5.30%	(5.22% to 5.38%)	4.00%	(3.94% to 4.05%)	1.33	(1.30 to 1.35)	1.30%	(1.20% to 1.40%)
Chronic obstructive pulmonary disease	4.66%	(4.58% to 4.74%)	1.42%	(1.38% to 1.45%)	3.29	(3.19 to 3.38)	3.24%	(3.16% to 3.33%)
Dementia	1.32%	(1.28% to 1.36%)	0.87%	(0.84% to 0.90%)	1.52	(1.45 to 1.58)	0.45%	(0.40% to 0.50%)
Depression	16.93%	(16.80% to 17.06%)	11.13%	(11.02% to 11.23%)	1.52	(1.50 to 1.54)	5.80%	(5.64% to 5.97%)
Diabetes mellitus	10.14%	(10.03% to 10.25%)	5.15%	(5.08% to 5.21%)	1.97	(1.94 to 2.00)	4.99%	(4.86% to 5.12%)
Epilepsy	1.06%	(1.02% to 1.09%)	0.62%	(0.60% to 0.65%)	1.70	(1.61 to 1.78)	0.43%	(0.39% to 0.48%)
Heart failure	1.70%	(1.66% to 1.75%)	0.95%	(0.92% to 0.98%)	1.79	(1.72 to 1.86)	0.75%	(0.70% to 0.81%)
Hypertension	20.85%	(20.69% to 21.01%)	15.84%	(15.73% to 15.96%)	1.32	(1.30 to 1.33)	5.00%	(4.80% to 5.20%)
Severe mental illness (psychosis, schizophrenia or bipolar affective disorder)	1.67%	(1.63% to 1.72%)	0.61%	(0.59% to 0.64%)	2.72	(2.60 to 2.85)	1.06%	(1.01% to 1.11%)
Obesity	13.96%	(13.84% to 14.08%)	7.46%	(7.38% to 7.55%)	1.87	(1.84 to 1.90)	6.50%	(6.35% to 6.64%)
Peripheral arterial disease	1.35%	(1.30% to 1.39%)	0.52%	(0.50% to 0.54%)	2.58	(2.46 to 2.72)	0.83%	(0.78% to 0.87%)
Rheumatoid arthritis	0.99%	(0.95% to 1.02%)	0.72%	(0.70% to 0.75%)	1.37	(1.30 to 1.44)	0.26%	(0.22% to 0.31%)
Stroke and transient ischaemic attack	2.89%	(2.83% to 2.95%)	1.97%	(1.93% to 2.01%)	1.46	(1.42 to 1.51)	0.91%	(0.84% to 0.99%)

IMD, Index of Multiple Deprivation; RR, rate ratio.

### Variation in prevalence by age and sex

**Figure 2:**
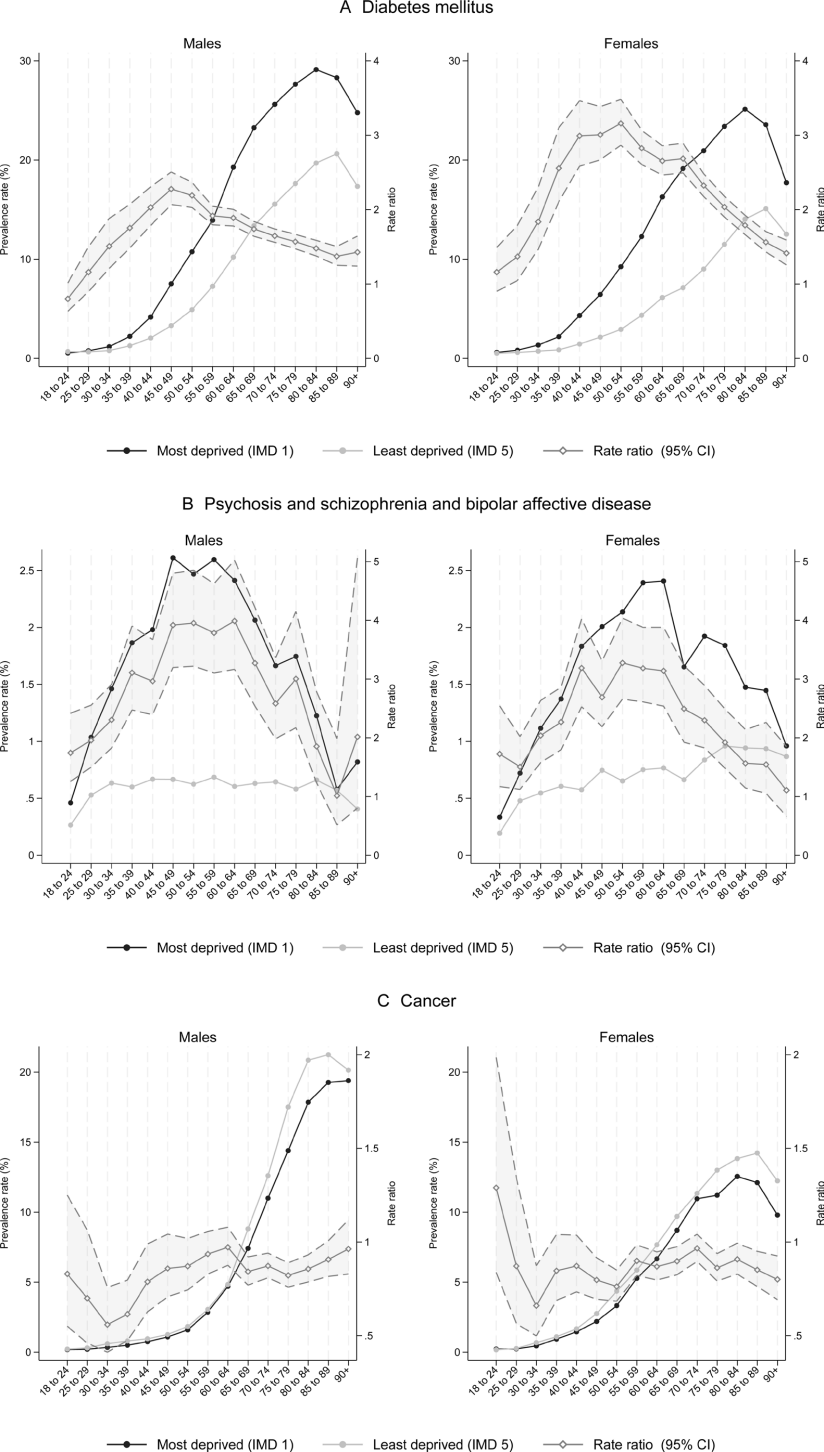
Prevalence rates of diabetes (A), severe mental illness (B) and cancer (C) in the most and least deprived fifth of the population, by age group and sex. IMD, Index of Multiple Deprivation.

Age-sex-deprivation-specific prevalence rates for each of the 17 conditions are reported in tabular and graphical form in the online supplemental material. Graphical representations of three conditions (diabetes, serious mental illness and cancer) illustrating typical prevalence patterns are presented in figure 2.

With respect to age, prevalence followed two main patterns: (1) an s-shaped distribution, with increasing prevalence between young adulthood and old age—in some cases with a small decline in the oldest age groups (eg, diabetes—figure 2A); (2) an n-shaped distribution, with increasing prevalence between young adulthood and middle age, followed by declining prevalence into the oldest age groups (eg, serious mental illness—figure 2B). With respect to sex, cardiovascular conditions, cancer, diabetes, serious mental illness and COPD were more prevalent overall in males, and rheumatoid arthritis, obesity, depression, dementia, chronic kidney disease and asthma were more prevalent overall in females, but relative prevalence varied by age group.

Inequalities in prevalence varied substantially with age and sex. For most conditions, relative inequalities—measured by the prevalence RR between the most and least deprived groups—followed an n-shaped distribution, increasing with prevalence between young adulthood and middle age before declining, with different inflection points for different conditions (see, eg, diabetes (figure 2A) and serious mental illness (figure 2B)). For some conditions (cancer (figure 2C), rheumatoid arthritis, dementia and atrial fibrillation), the distribution was flatter, with similar prevalence RRs across most age groups.

For most conditions, relative inequalities were greater in females than males, for example, the prevalence RR for diabetes peaked at 2.3 in males and 3.2 in females. Relative inequalities were greater for males than females for two conditions (serious mental illness and depression) and RRs were broadly similar in males and females for five conditions (cancer, chronic kidney disease, dementia, epilepsy and rheumatoid arthritis). Across all conditions, the highest peak relative inequality (excluding age-sex-prevalence combinations with very low numbers) was for peripheral arterial disease, with a RR of 7.6 for females aged 55–59.

In absolute terms, inequalities also tended to be greater for females than males and to increase from early to late adulthood—later than for relative inequalities—before declining at the extremes of age. The highest peak absolute inequality was for hypertension, with a rate difference of 1390 per 10 000 for females aged 65–69.

## Discussion

### Main findings

We used routinely collected EPCRs to estimate socioeconomic variations in recorded prevalence of 17 common long-term conditions in England, finding large and clinically significant differences in all cases. For 16 conditions, age-sex-adjusted prevalence was higher in patients living in more deprived areas, with relative rates between the most and least deprived fifth of areas ranging from 1.04 times higher for atrial fibrillation to 3.3 times higher for COPD. Only cancer was more prevalent in patients living in less deprived areas, but note that cancer covers a wide range of conditions; relative prevalence rates for specific malignancies will vary.^24^

Inequalities were small in the youngest age groups for whom most long-term conditions are uncommon but became substantial in middle age as these conditions started to emerge. For some conditions—for example, cardiovascular conditions—prevalence continued to increase in the oldest age groups, whereas for others—such as depression and serious mental illness—prevalence fell with age. Regardless of the underlying pattern of prevalence, inequalities increased for most conditions between early adulthood and middle age before falling in the oldest age groups. Inequalities were greater in females than males for most conditions, which may reflect gender differences in a range of mechanisms, such as health behaviour, risk exposure or discrimination.^25 26^

### Measuring inequalities

We provided different perspectives on socioeconomic variation in disease burden to address different policy priorities. For example, data on observed disease prevalence by population subgroup are needed to determine how policy and public health interventions should be targeted to reduce health inequalities in the existing population. In contrast, prevalence rates standardised to a common age-sex distribution are concerned with hypothetical populations not observed in practice, which limits their usefulness for service design or clinical decision-making but provides important information on the relative risk of disease faced by different population groups.

The difference between observed and age-sex-standardised rates is substantial. Age-sex-standardised prevalence rates were higher in more deprived population groups compared with less deprived populations for most conditions. These findings are in line with previous work on the prevalence of atrial fibrillation,^27^ chronic kidney disease,^28^ COPD,^29^ depression,^30^ diabetes,^18 31^ heart failure,^32^ severe mental illness,^33^ obesity^34^ and other health conditions in the English general population, which used a variety of data sources and equity stratifiers. However, this does not reflect what practitioners observe in daily practice, where a third of the 17 diseases analysed in this study are more commonly observed among less deprived members of society, who are, on average, older and live longer lives.

We also provide age-specific and sex-specific prevalence rates for each condition and estimates of relative inequalities between people living in the least and most deprived fifth of areas in England. For some conditions—for example, stroke—prevalence is higher in less deprived groups for some age-sex categories and higher in more deprived groups for others. For conditions with consistently higher prevalence in more deprived groups, prevalence RRs may vary by up to seven times between different age-sex groups; overall estimates of inequalities ignore this wide variation.

### Strengths and limitations

We used a large, representative and quality-assured database of EPCRs drawn from GP practices in England that collect clinical information prospectively during patient management activities. These data are linked to a comprehensive and granular measure of area deprivation through individual patient postcodes, allowing precise prevalence rates to be calculated for different population subgroups. Our study also exploits external standards for disease identification implemented as part of a national pay-for-performance programme with strong financial incentives, limiting the scope for systematic biases in how different healthcare providers diagnose and record conditions. The QOF business rules provide practices with a standard set of diagnostic codes for incentivised conditions, which have a very high degree of accuracy in terms of correctly identifying patients as having the relevant condition, and a high but lower degree of accuracy in terms of completeness (ie, practices may fail to apply diagnostic codes to some patients with the condition^35^).

The study also has several limitations. It is descriptive, so although it provides evidence for associations between area-level deprivation and prevalence of common long-term conditions, it does not establish causation at the individual level. There is, however, extensive evidence that components of the IMD, such as income and education, are powerful determinants of health (causation) and also that poor health can lead to lower income and other markers of socioeconomic status (health selection), with causation becoming increasingly influential with advancing age.^36^ Additionally, our study focuses on the adult population, limiting comparability to other nationally reported disease registers, which may include the 21% of the English population who are 17 years or younger.^23^ Since most study conditions are rare in children and adolescents, our estimates of disease prevalence in the adult population are expected to be larger than age-unrestricted prevalence rates. Although CPRD data include primary care activity (diagnosis, prescribing, laboratory results and referrals) and linkages to secondary care and other administrative datasets, accuracy with respect to prevalence estimates could potentially be improved with additional linkages and the use of advanced phenotype algorithms.

Our study focused on diagnosed and recorded disease prevalence and does not (directly) measure population disease burden or unmet need. Patients do not generally encounter access charges for NHS primary care, so there are no direct financial barriers which could lead to systematic undercounts in more deprived populations and socioeconomic distributions in diagnosis closely match distributions in self-reported burden of disease for QOF conditions such as diabetes.^30^ However, non-financial barriers to access^37^ result in underdiagnosis in more deprived groups for other conditions, such as angina^30^; for these conditions, measures of inequality based on recorded diagnosis are likely to underestimate inequalities in population prevalence. Burden of disease is also affected by quality of care, which is socioeconomically patterned.^38^ The study also focused on single conditions and therefore overlooks interactions between conditions and the social patterning of multimorbidity,^13^ which poses a major and growing challenge to health services.^39^ Our detailed approach to single conditions could be extended to examine the social patterning of combinations of conditions across the lifecourse.

## Conclusions

Our study followed a common data extraction and analysis protocol to study the prevalence of a range of long-term health conditions in EPCRs, generating a catalogue of disease estimates of comparable quality. This catalogue can aid further modelling, for example, to inform resource allocation decisions or facilitate distributional cost-effectiveness analysis.^40^ In addition, we provide estimates of disease prevalence by 5-year age band-sex-IMD quintile combination in the online supplemental material, which can be combined with population projections to forecast changes in disease patterns over time due to demographic change, or to model the expected number of patients at subnational level.

## Supplementary material

10.1136/jech-2024-223553 online supplemental file 1

## Data Availability

Data may be obtained from a third party and are not publicly available.
